# Pattern of Medico-legal Cases among Pediatric Patients Presenting to the Emergency Department of a Tertiary Care Center in Nepal: An Observational Study

**DOI:** 10.31729/jnma.v63i2091.9234

**Published:** 2025-11-30

**Authors:** Bishnu Bhujel, Minani Gurung, Lisasha Poudel, Anima Bhandari, Bibek Rajbhandari

**Affiliations:** 1 Paropakar Nursing Campus Kupondole, Lalitpur, Nepal; 2 Kathmandu University School of Medical Sciences, Dhulikhel, Kathmandu, Nepal; 3 Institute for Implementation Science and Health, Kathmandu, Nepal; 4 Maharajgunj Medical Campus, Kathmandu, Nepal

**Keywords:** *injury prevention*, *nepal*, *pediatric medico-legal cases*, *road traffic accidents*, *suicide and self-harm*

## Abstract

**Introduction:**

Pediatric medico-legal cases, encompassing injuries and other unnatural events, pose a significant public health burden, particularly in low- and middle-income countries like Nepal. Lack of comprehensive, localized data on the spectrum of these cases presenting to emergency departments hinders the development of targeted prevention and management strategies. This study aimed to describe the pattern of medico-legal cases among pediatric patients at a tertiary care center in Nepal.

**Methods:**

A retrospective cross-sectional study was conducted by reviewing the medical charts of pediatric patients (under 18 years) registered as medico-legal cases in the Emergency Department of Tribhuvan University Teaching Hospital from December 2022 to January 2023. Data on demographics, case type, and clinical details were extracted and analyzed using descriptive statistics.

**Results:**

Out of 2,936 total medico-legal cases, 297 (10.11%) were pediatric cases. Based on types of incidents, there were 109 (36.70%) road traffic accidents, 68 (22.89%) suicide and self-harm, 47 (15.82%) falls, and 46 (15.49%) physical assaults. Adolescents aged 14-17 years accounted for 147 (49.49%) cases, and males accounted for 182 (61.15%) cases. There were 57 (83.8%) suicide attempts with poisoning, and 10 (52.63%) accidental poisonings with pesticides. Males accounted for 77 (70.64%) of road traffic accidents and 34 (73.91%) of physical assaults, while females accounted for 39 (57.35%) of suicide/self-harm cases.

**Conclusions:**

Road traffic accidents and suicide/self-harm are the leading causes of pediatric medico-legal presentations in this tertiary care setting. The distinct patterns observed across different age and gender groups underscore the need for targeted, evidence-based interventions in injury prevention, mental health support, and public safety to protect this vulnerable population in Nepal.

## INTRODUCTION

Pediatric injuries and unnatural events represent a significant global public health concern, causing significant morbidity, disability, and mortality.^1-6^ This burden is higher in low- and middle-income countries like Nepal, where rapid urbanization and limited safety awareness increase risks for children.^7-11^ In hospital emergency departments, such incidents are categorized as medico-legal cases (MLCs) in any condition or injury from suspected external or non-natural causes, including road traffic accidents, falls, poisonings, assaults, and self-harm.^12-16^ Managing pediatric MLCs is important, extending beyond immediate medical treatment to fulfill legal and child protection obligations.^17^ However, there is a lack of comprehensive, localized data on the spectrum of pediatric MLCs in Nepal, limiting the development of evidence-based clinical guidelines and preventive strategies.^18,19^ This study aims to address this gap by analyzing MLCs in a pediatric emergency setting. Its objective is to describe the types of medico-legal cases and their variation by age and gender, providing evidence to promote safer environments and better medical responses for children in Nepal.

## METHODS

A retrospective cross-sectional study was conducted to assess the pattern of medico-legal cases among pediatric patients presenting to the Emergency Department of Tribhuvan University Teaching Hospital (TUTH), Nepal. TUTH is a tertiary care referral center and one of the largest tertiary hospitals in the country, serving a diverse patient population from Kathmandu and beyond.

Ethical approval for this study was obtained from the Institutional Review Committee (IRC) of the Institute of Medicine, TUTH (Reference No. 194). As a retrospective chart review, the study posed minimal risk to patients. All patient data was anonymized, and confidentiality was maintained throughout the data collection and analysis process.

We conducted a retrospective review of medical charts for the period from December 2022 to January 2023. The study population included all patients under the age of 18 years who visited the Emergency Department and were registered as a medico-legal case during this period. All medical records of patients under 18 years of age that were officially registered as medico-legal cases in the hospital’s medico-legal record book maintained by the TUTH Emergency Department during the study period were included. Records that were incomplete or illegible were excluded from the final analysis.

At TUTH, a case is designated as medico-legal by the examining physician in the Emergency Department based on the nature of the illness or injury and the history provided. Cases that are typically registered as medico-legal include a wide range of situations where legal implications may arise from medical circumstances. These generally encompass all forms of trauma resulting from suspected physical assault, as well as all road traffic accidents (RTAs). Additionally, any known or suspected cases of poisoning whether accidental, suicidal, or homicidal in nature are categorized as medico-legal. Instances of alleged sexual assault also fall under this classification. Injuries sustained from falls that occur under suspicious circumstances must be treated as medico-legal cases. Furthermore, any cases brought in by the Nepal Police for examination and official reporting are automatically registered as medico-legal matters.

Once a case is identified as medico-legal, it is documented in a separate medico-legal register maintained by the Emergency Department. A detailed Medico-Legal Certificate (MLC) is prepared, which documents the patient’s history, examination findings, and the doctor’s professional opinion regarding the nature, cause, and manner of the injury or event.

Following this initial medical documentation, all patients with a medico-legal tag become subject to investigation by Nepal Police personnel stationed at the hospital. Every such case is reported to the Maharajgunj Police Station. In instances of a patient’s death within the Emergency Department, a mandatory postmortem examination is conducted. Similarly, if patients are referred to another hospital or choose to leave against medical advice (LAMA), these situations are also reported to the on-duty police and may prompt further inquiry.

For the purpose of this study, patients were identified for inclusion if their case was officially recorded in the aforementioned medico-legal register.

Data was extracted from the official medico-legal registers and the corresponding patient medical charts from the hospital’s Medical Record Section. A pre-designed, structured data collection proforma was used to extract specific variables relevant to the study. These included demographic details such as age, which was categorized into groups of 0-4, 5-9, 10-13, and 14-17 years, along with gender (male or female) and ethnicity. Clinical details encompassed the outcome of each case classified as discharged, death, referred, leave against medical advice, or no record and the Emergency Room (ER) triage category assigned upon admission, which included red, yellow, and green codes. Case details were also documented, focusing on the primary type of medico-legal case, such as physical assault, poisoning, road traffic accident (RTA), suicide or self-harm, fall, or others, along with further specifics where available, including the method of suicide, type of poison, and nature of physical assault.

The collected data was first entered into a Microsoft Excel spreadsheet (Version 16.0) and subsequently cleaned and crosschecked for inconsistencies or errors. The cleaned dataset was then imported into the Statistical Package for the Social Sciences (SPSS) Version 25.0 for analysis. Descriptive statistics were used to summarize the data; frequencies and percentages (n (%)) were calculated for all categorical variables.

## RESULTS

Out of 2,936 total medico-legal cases recorded during the study period, 297 (10.11%) involved pediatric patients. The distribution of pediatric medico-legal cases by type is presented in Table 1. Road Traffic Accidents (RTAs) accounted for 109 (36.70%) cases, suicide and self-harm for 68 (22.89%), falls for 47 (15.82%), physical assaults for 46 (15.49%), accidental poisoning for 19 (6.40%), and other causes such as accidental burns, electrocution, and sexual assault for 8 (2.69%) cases (Table 1).

**Table 1:** Type of medico-legal type (n=297).

Variables	n (%)
Physical Assault	46 (15.49)
Poisoning (accidental)	19 (6.40)
Road Traffic Accidents (RTAs)	109 (36.70)
Suicide and self-harm	68 (22.89)
Fall	47(15.82)
Others (Electroculation/accidental burn/Sexual Assault)	8(2.69)

**Table 2:** Characteristics of specific medico-legal cases (n=297).

Medico-legal cases	n(%)
**Method of Suicide (n=68)**	
Cutting arteries or veins	6(8.96)
Hanging	5(7.46)
Poisoning	57(83.8)
**Types of Poisoning for suicide (n=57)**	
Carbomonoxide poisoning	1(1.75)
Chemical substance	7 (12.28)
Drug Overdose	9(15.79)
Pesticides	40(70.18)
**Types of Physical Assault (n=46)**	
Blunt Force Trauma	38(82.61)
Sharp Force Trauma	6(13.04)
Strangulation	2(4.35)
**Poisoning (Accidental) (n=19)**	
Pesticides	10(52.63)
Chemical substances	6(31.58)
Medicines	2(10.53)
Carbon monoxide poisoning	1(5.26)

*Blunt force trauma includes injuries caused by impact with hard objects such as sticks, stones, fists etc. *Sharp force trauma includes injuries inflicted by sharp objects such as knives, sickles (khurpa), or broken glass.

**Table 3:** Characteristics of pediatric cases (n=297).

Characteristic	n (%)
**Age Category (in years)**	
0-4	21(7.07)
5-9	55(18.52)
10-13	74(24.92)
14-17	147(49.49)
**Gender**	
Female	115(38.85)
Male	182(61.15)
**Ethnicity**	
Brahmin/Chettri	106(35.69)
Dalit	30(10.10)
Gurung/Rai/Magar/Sherpa/Lama	111(37.37)
Madhesi	28(9.43)
Muslim	4(1.35)
Newar	18(6.06)
**Fate/Outcome**	
Death	10(3.36)
Discharged	34(11.44)
Leave Against Medical Advice	27(9.09)
No record	8(2.69)
Referred to other department for treatment	218(73.4)
**ER Triage**	
Green	177(61.67)
Red	116(39.05)
Yellow	4(1.39)

**Table 4:** Medical-legal cases among gender and age group (n=297).

Age Group	Physical Assault	Poisoning (Accidental)	Road Traffic Accident	Suicide and Self-Harm	Falls
0-4	2(4.35)	0(0.00)	13(11.93)	0(0.00)	3(6.38)
5-9	5(10.87)	3(15.79)	29(26.61)	3(4.41)	15(31.91)
10-13	21(45.65)	4(21.05)	22(20.18)	9(13.24)	17(36.17)
14-17	18(39.13)	12(63.16)	45(41.28)	56(82.35)	12(25.53)
**Gender**					
Female	12(26.09)	11(57.89)	32(29.36)	39(57.35)	20(42.55)
Male	34(73.91)	8(42.11)	77(70.64)	29(42.65)	27(57.45)

Among the 68 cases of suicide and self-harm, poisoning occurred in 57 (83.8%) cases, cutting arteries or veins in 6 (8.96%) cases, and hanging in 5 (7.46%) cases. Of the 57 poisoning cases, pesticides were involved in 40 (70.18%) cases, drug overdose in 9(15.79%) cases, and other chemical substances in 7 (12.28%) cases. Among the 46 cases of physical assault, blunt force trauma was recorded in 38 (82.61%) cases, sharp force trauma in 6 (13.04%) cases, and strangulation in 2 (4.35%) cases. In 19 cases of accidental poisoning, pesticides were reported in 10 (52.63%) cases, chemical substances in 6 (31.58%) cases, medicines in 2 (10.53%) cases, and carbon monoxide poisoning in 1 (5.26%) case (Table 2).

There were 147 (49.49%) patients were aged 14-17 years, and 182 (61.15%) were male. The ethnic distribution included Janjati 111 (37.37%) and Brahmin/Chettri 106 (35.69%). Upon arrival at the emergency department, 116 (36.93%) children were triaged as ‘Red,’ and 10 (3.36%) children died as the final outcome in the emergency room (Table 3).

Suicide and self-harm were reported in 56 (82.35%) patients aged 14-17 years. Physical assault was recorded in 21 (45.65%) children aged 10-13 years. Accidental poisoning was observed in 12 (63.16%) patients aged 14-17 years, road traffic accidents in 45 (41.28%) patients aged 14-17 years, and falls in 17 (36.17%) patients aged 10-13 years and 15 (31.91%) patients aged 5-9 years. Among males, 34 (73.91%) had physical assault, and 77 (70.64%) had road traffic accidents. Among females, 39 (57.35%) had suicide and self-harm, and 11 (57.89%) had accidental poisoning (Table 4).

## DISCUSSION

This study provides a detailed analysis of the pattern of pediatric medico-legal cases (MLCs) at a major tertiary referral hospital in Nepal, revealing a landscape dominated by preventable injuries and urgent public health challenges. Our principal findings indicate that Road Traffic Accidents (RTAs) are the most frequent cause for pediatric MLC presentations, followed by an alarming rate of suicide and self-harm. These incidents disproportionately affect adolescents, with distinct and significant patterns emerging when stratified by age and gender. Male adolescents are particularly vulnerable to trauma-related incidents, while female adolescents are overrepresented in cases of self-harm.

The majority of patients were adolescents aged 14-17 years (49.49%) and male (61.15%). A similar study conducted by Mina et al. (2024) in India reported that the majority of victims (29%) were aged 16-18 years, followed by 24.36% in the 11-15 years group.^15^ A similar study in India, conducted by Mrunalini et al., also showed a majority among males.^20^ Activities such as increased outdoor engagement and a tendency to experiment with dangerous actions like unsafe driving or substance use render this group more susceptible to both accidental injuries and violence.^21,22^

The finding that RTAs account for over a third (36.70%) of all pediatric MLCs aligns with data from other low- and middle-income countries (LMICs), where injuries are a leading cause of childhood morbidity and mortality.^23^ This high prevalence often seen in Kathmandu is likely a direct consequence of rapid and often unregulated urbanization, a surge in vehicle density, and infrastructure that has not kept pace with the need for pedestrian safety, such as protected walkways and safe road crossings. The concentration of RTA cases among male adolescents aged 14-17 may reflect a combination of greater mobility, higher engagement in risk-taking behaviors, and increased use of twowheeled vehicles, underscoring a critical target group for road safety interventions.

Perhaps the most alarming finding of this study is the high prevalence of suicide and self-harm, which constitutes the second leading cause of pediatric MLCs (22.89%). This highlights a severe and growing mental health crisis among Nepalese youth. The concentration of these cases among adolescents, particularly females, is consistent with global trends.^24-26^ While suicide is a massive public health challenge worldwide, with 746,000 deaths (95% UI 692,000-800,000) reported globally in 2021,^26^ including 519,000 males and 227,000 females, the findings also point to specific local stressors. These may include immense academic pressure, sexual assault, family conflict, and limited access to mental health support.^27^ The predominant method identified poisoning (83.8%) and the specific agent pesticides (70.18%) present an urgent and clear opportunity for prevention. The ready availability of highly lethal agricultural chemicals in both rural and urban households serves as a critical “means” that transforms impulsive acts of distress into fatal or near-fatal events. This finding strongly suggests that policies restricting access to the most toxic pesticides could be a highly effective, life-saving intervention.

The patterns observed in other trauma categories further illuminate age-specific vulnerabilities. The higher incidence of falls among younger children (5-13 years) likely corresponds to developmental stages of increased physical activity and exploration, possibly compounded by unsafe living environments, such as multi-story buildings lacking adequate safety features.^28^ Meanwhile, physical assault was observed in 15% of children. The peak of physical assaults among children aged 10-13 years raises concerns about violence in various settings, including schools (bullying), the community, or within the home, warranting further investigation into child protection issues.

The implications of these findings are manifold. For clinical practice, the high volume and severity of these cases, with over a third of patients triaged as ‘Red’ necessitate specialized training for emergency department staff in pediatric trauma and MLC management. Furthermore, the high rate of self-harm mandates the integration of mental health services into emergency care, including routine screening and a clear referral pathway to psychiatric support for at-risk youth.

From a public health policy perspective, this data serves as a compelling call to action. It provides the evidence base needed to advocate for multi-sectoral interventions, including the enforcement of road safety laws, the development of safer urban infrastructure, and, most urgently, the implementation of national policies to regulate the sale and storage of highly hazardous pesticides. For future research, prospective, multicenter studies are needed to create a more comprehensive national picture of pediatric injuries. Qualitative studies are also essential to understand the underlying social and psychological drivers of the adolescent mental health crisis in Nepal.

There are a few limitations of this study. As a single-center, retrospective analysis, its findings may not be fully generalizable to the entire country, particularly rural regions where both risks and healthcare access differ. The reliance on existing medical records means our analysis is constrained by the quality and completeness of that documentation, a limitation underscored by the significant amount of missing data on patient outcomes.

## CONCLUSION

This study confirms that preventable incidents chiefly road traffic accidents and acts of self-harm are the primary drivers of pediatric medico-legal presentations at a major Nepalese hospital.

## Data Availability

The data are available from the corresponding author upon reasonable request.
